# Traditional East Asian Herbal Medicine for Amyotrophic Lateral Sclerosis: A Scoping Review

**DOI:** 10.1155/2021/5674142

**Published:** 2021-12-06

**Authors:** Won Joo Suh, Yuna Seo, Chul Jin, Seung-Yeon Cho, Seong-Uk Park, Woo-Sang Jung, Sang-Kwan Moon, Jung-Mi Park, Chang-Nam Ko, Seungwon Kwon, Ki-Ho Cho

**Affiliations:** Department of Cardiology and Neurology, College of Korean Medicine, Kyung Hee University, Seoul 02447, Republic of Korea

## Abstract

This study aimed to analyze and summarize the existing evidence regarding herbal medicine treatments for amyotrophic lateral sclerosis (ALS). Studies on herbal medicine treatment in patients with ALS were searched within English, Chinese, Japanese, and Korean databases up to July 31, 2021. In the selected studies, we collected the following information: the first author, year of publication, country, language, study methodology, sample size, demographic characteristics of the study participants, disease duration, diagnostic criteria, treatment method, treatment periods, evaluation tools, results, and side effects. The organized data were classified and analyzed narratively. This study included 59 studies. The first clinical study on the effect of herbal medicine was published in 1995; moreover, most studies were conducted in China. Among the 59 selected studies, 47.5% were observational studies, including case reports and case series. Moreover, there was one meta-analysis. The El Escorial criteria were the most commonly used diagnostic criterion for ALS; moreover, the ALS functional rating scale was the most common evaluation tool. Buzhongyiqitang, Sijunzitangjiawei, and Jianpiyifeitang were the most commonly used herbal medicines, with anti-inflammatory, protein aggregation, and anti-oxidant effects. There remain evidence of gaps in the effectiveness of herbal medicine for ALS. To allow effective treatment of patients with ALS using herbal medicine, large-scale and rigorously designed high-quality clinical studies should be performed.

## 1. Introduction

Amyotrophic lateral sclerosis (ALS) is a representative motor neuron disease involving invasion of the upper and lower motor neurons. The cerebral cortex, brainstem, and spinal motor nerves are selectively invaded, which results in gradual muscle weakness and death from respiratory muscle paralysis within 2–4 years [1]. The disease was first reported by Charles Bell in 1824 and was termed ALS in 1874 by Jean-Martin Charcot, who linked the symptoms to neurological problems [2]. ALS is also termed Lou Gehrig's disease after a famous baseball player, Lou Gehrig, who suffered from the disease in 1939 [3].

Approximately 90% and 10% of ALS cases are sporadic and familial, respectively. However, the ALS prevalence rate in South Korea remains unclear. A recent study reported an ALS incidence of 0.6 to 3.8/100,000 per year, with men having a slightly higher prevalence rate than women. The age of onset for sporadic and familial ALS is 58–63 years and 47–52 years, respectively; moreover, the incidence rate rapidly decreases after the age of 80 years. Familial ALS is mainly associated with mutations in genes, including superoxide dismutase 1 and transactive response DNA-binding protein. The cause of sporadic ALS is unknown; however, it involves environmental and genetic risk factors. The underlying pathogenesis of motor neuron degeneration in ALS is protein aggregation and glutamate-induced excitatory toxicity. Neuroinflammation is crucially involved in motor neuron cell death in ALS by accelerating the disease progression; moreover, oxidative stress contributes to motor neuron damage. However, details regarding the underlying mechanism remain to be elucidated [1, 4–6].

Currently, the Food and Drug Administration-approved drugs for ALS treatment are riluzole and edaravone. Riluzole is an anti-glutamate drug that can extend the lifespan by 2-3 months, while edaravone is an anti-oxidant drug primarily used for post-stroke recovery. However, these drugs cause side effects, including diarrhea, fever, nausea, vomiting, dizziness, and fatigue; moreover, there is currently no treatment that can fundamentally cure or stop ALS progression [7].

Therefore, patients with ALS and their families can consider complementary alternative medicine (CAM) for ALS treatment. A Chinese study [8] reported that 99% of patients with ALS underwent integrative therapy (IT); moreover, a recent South Korean study [9] reported that every patient who participated in the survey received CAM treatment. In a German questionnaire study [10], 54% of patients with ALS reported having received CAM treatment. These findings suggest that CAM treatment is often used in both Western and Eastern societies.

Herbal medicine is a key CAM therapy; moreover, it may serve as an alternative when no fundamental treatment is available. In particular, in East Asian countries such as China, Korea, and Japan, traditional East Asian herbal medicines, which reflect regional characteristics and share basic concepts, have been applied to the treatment of various diseases. Therefore, in these countries, there is a constant demand for treatment using traditional East Asian herbal medicines for intractable neurological diseases, such as ALS, that cannot be significantly effective with conventional therapies alone [8, 9]. In response to that demand, research using traditional East Asian herbal medicines continues. As a result, there are currently active studies on whether herbal medicines with anti-glutamate, protein aggregation inhibition, anti-inflammatory, and anti-oxidant properties can be used for ALS treatment. However, there remains a lack of evidence regarding herbal medicine treatment for ALS [4]. Therefore, this study aimed to assess and analyze evidence regarding the effects of herbal medicine for ALS treatment and identify future research directions.

## 2. Materials and Methods

This was a scoping review of clinical studies on herbal medicine for ALS treatment. A scoping review is a research method that rapidly maps key concepts, sources, and evidence types supporting the research area. This helps identify gaps in evidence by determining areas with insufficient research areas based on conclusions from the literature regarding the status of overall research activity. This study was conducted in sequence following the scoping review guidelines suggested by the Joanna Briggs Institute and based on the checklists of Preferred Reporting Items for Systematic Reviews and Meta-Analyses extension for scoping review (PRISMA-ScR) [11, 12].

### 2.1. Literature Selection Criteria

#### 2.1.1. Research Questions

This study aimed to analyze studies on herbal medicine treatment for ALS to assess the treatment effects, evidence regarding the effects, and the type of future studies required for further evidence.

#### 2.1.2. Research Design Type

This study aimed to examine and connect the contents regarding the research questions. Therefore, we included various types of clinical studies, including case reports, retrospective and prospective observational studies, clinical trials, narrative reviews, systematic reviews, and meta-analyses.

#### 2.1.3. Research Subject Type

This study included patients with confirmed ALS. We included studies that reported ALS diagnosis without specifying the diagnostic criteria. However, studies that presumed an ALS diagnosis were excluded. Moreover, we excluded studies on ALS comorbid with other diseases, including myasthenia gravis and progressive muscular atrophy. Additionally, we excluded studies on cell and animal models that were not conducted on humans.

#### 2.1.4. Intervention Type

We included studies that provided oral herbal medicine treatment as an intervention, regardless of the dosage form. We included typical types of herbal prescriptions, including traditional Chinese medicine (TCM), traditional Korean medicine (TKM), and Japanese Kampo medicine, which have been mainly used as traditional medicine in Northeast Asian countries. Moreover, we included studies that used herbal medicine in combination with other treatment methods or that compared the effects of herbal medicine with those of other treatments. We excluded studies that assessed vitamins and other food supplements, as well as herbal products and extracts that are not commonly used in traditional medicine in Northeast Asian countries, including South Korea, China, Japan, and Taiwan. Moreover, medicinal herbs that are currently not being used, including bear gall bladders, were excluded.

#### 2.1.5. Research Measurement Type

All study results, including changes in clinical symptoms, measurement of various evaluation tools, total effective rate (TER), and the average symptom score after herbal treatment, were assessed. We excluded studies that did not state the effects of herbal medicine treatments.

### 2.2. Literature Search Strategy

We used the following eight online databases: Medline, Embase, Cochrane, Scopus, Chinese National Knowledge Infrastructure, Citation Information by National Institute of Informatics, National Digital Science Library, and Oriental Medicine Advanced Searching Integrated System. The last search date was until July 31, 2021. The search terms used in this study were based on MeSH terms of studies on identical topics; moreover, natural words were selected with respect to various expressions and similar words used in different studies. Based on the Medline search strategy, we converted search terms to include the characteristics of each database. The search expressions are shown in Appendix 1. There was no language restriction to allow review of a wide range of literature; moreover, gray literature, including theses and reports, was included. Endnote X9 was used to manage the studies.

### 2.3. Data Analysis

First, the title and abstract of each study were reviewed; subsequently, the full texts of studies that met the selection criteria were reviewed. Each paper was reviewed regardless of the original language, and when the professional translation was required, a translation expert in the relevant language was consulted. Two researchers (Suh WJ and Seo YN) independently reviewed the literature. In cases of disagreement, the studies were reviewed together to reach a consensus. When a consensus could not be reached, a third researcher (Kwon S) was consulted.

The selected studies were reviewed; moreover, the contents of each study were organized based on the data collection form. The form included the first author, year of publication, country, language, research methods, number of study participants, demographic characteristics of the participants, study duration, diagnostic criteria, treatment method, treatment duration, evaluation tools, outcomes, and side effects. The country was based on the location of the author's organization/institute; moreover, the language referred to the language used to write the manuscript. The research methodology was largely divided into observational studies, intervention studies, and literature studies based on the classification of research methods used by the National Institute for Health and Care Excellence, which were further divided into case reports, case series, prospective and retrospective observational studies, randomized control studies, narrative reviews, systematic reviews, and meta-analyses. Case reports describe the intervention and results of the patient cases. A single case was referred to as a single case report, while two or more cases were classified as a case series. Observational studies on natural interventions were divided into prospective and retrospective observational studies. Before-and-after studies compared the results before and after the interventions. Randomized control studies included clinical trial studies where participants were randomly assigned to an experimental or control group with a certain follow period for assessing specific outcomes. Systematic review studies referred to studies that used explicit and systematic methods to identify, evaluate, and summarize the literature using predefined criteria. If there was no explanation regarding the criteria, those studies were considered narrative review studies. Meta-analyses statistically combine and analyze the findings from at least two studies. Studies that performed meta-analysis after a systematic review of the literature were considered meta-analyses. Studies without a clearly specified methodology were assigned to the most appropriate group based on the study contents. Demographic characteristics of the study participants included sex and age. The diagnostic criteria used to confirm ALS are summarized. In case no diagnostic criteria were mentioned, we assessed the tests performed for diagnosis, excluding clinical symptoms. All evaluation tools used in the studies were reviewed; moreover, those that yielded meaningful results were separately summarized. Subsequently, the organized data were classified and analyzed based on the research methods.

## 3. Results

### 3.1. Literature Selection

**Figure 1:**
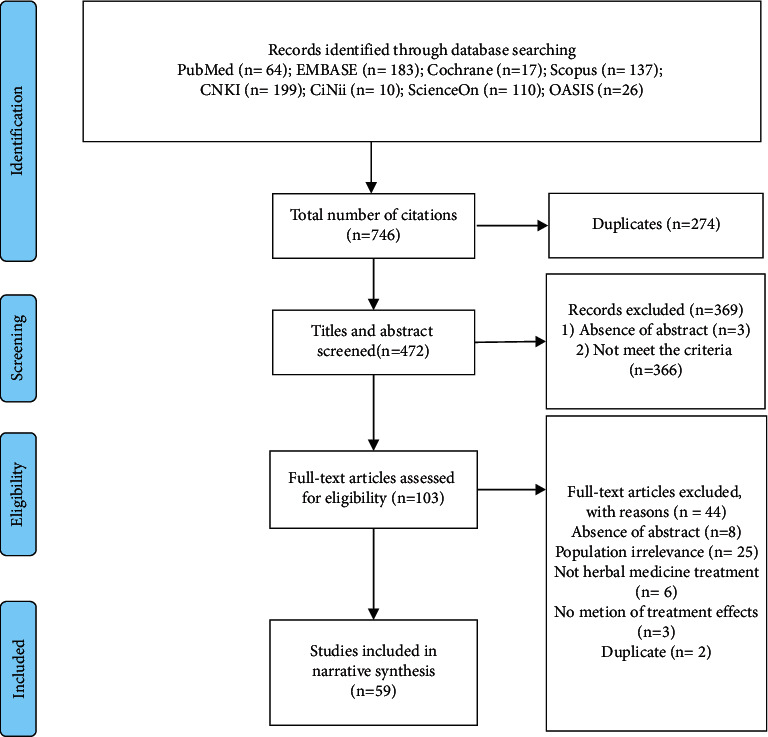
PRISMA flow chart of the study selection process.

**Figure 2:**
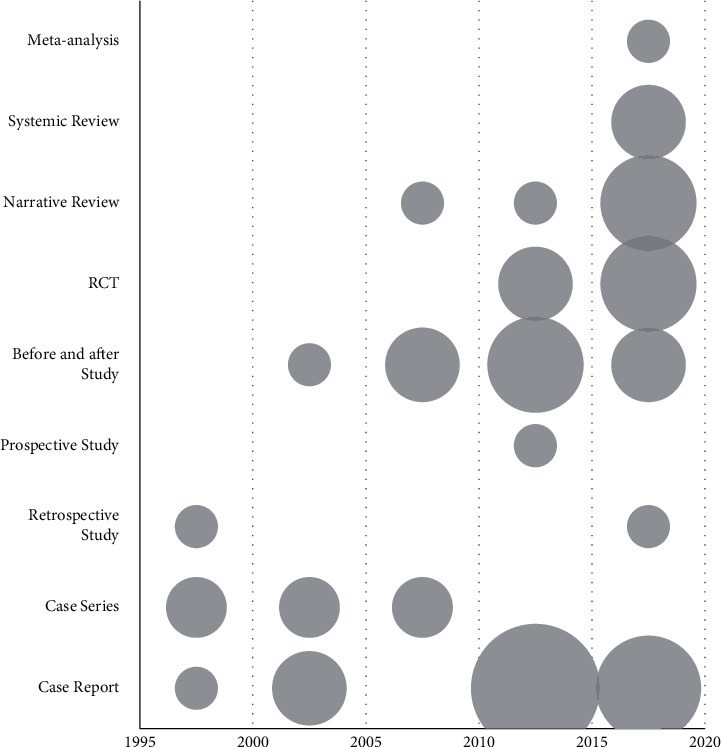
Distribution of literature status according to year. Bubble size: the number of corresponding literature. RCT: randomized controlled trial.

We searched 746 studies; among them, we reviewed the titles and abstracts of 472 studies after excluding 274 duplicates. In case the abstract was unavailable, the full text was reviewed. We excluded 3 studies without abstract and full text, as well as 366 articles that did not meet the selection criteria. The full text of the remaining 103 studies was reviewed; among them, 44 studies were excluded based on the selection criteria as follows: 8, 25, 6, 3, and 2 studies that did not have the full text, did not satisfy the type of study participants, did not use herbal medicine treatment, did not mention the effects after herbal medicine treatment, and were duplicates, respectively. Finally, 59 articles were included in the analysis (Figures 1 and 2).

### 3.2. General Characteristics of the Literature

To assess the general characteristics of the literature, the studies were cataloged and analyzed according to the year of publication, research method, country, and language. Tables 1 and 2 summarize the results.

#### 3.2.1. Year of Publication

Related studies were first published in 1995; moreover, approximately 73% of the included studies were published from 2010 to 2019 (Figure 2).

#### 3.2.2. Research Methods

All 59 studies were research papers with 28 observational studies [8, 13–39], 20 intervention studies [40–59], and 11 literature studies [4, 7, 60–68]. Among the 28 observational studies, 19, 6, 2, and 1 were case reports [13–31], case series [32–37], retrospective observational studies [38, 39], and prospective observational studies [8], respectively. Among the 20 intervention studies, 12 were before-and-after studies [40–51], while 8 were randomized control studies [52–59]. Among the 11 literature studies, 7, 3, and 1 were narrative review studies [7, 60–65], systematic review studies [4, 66, 67], and meta-analysis studies [68], respectively.

#### 3.2.3. Country of Study

All the studies were conducted in China and South Korea. There were 39 [4, 8, 13, 18, 19, 24–26, 29–34, 39–41, 44–58, 60–65, 68] and 20 [7, 14–17, 20–23, 27, 28, 35–38, 42, 43, 59, 66, 67] studies conducted in China and South Korea, respectively.

#### 3.2.4. Language

Regarding the language, 34, 14, and 11 studies were published in Chinese [13, 18, 19, 24–26, 30–34, 39–41, 44–53, 55–57, 60–65, 68], Korean [14–17, 22, 23, 27, 36–38, 42, 43, 66, 67], and English [4, 7, 8, 20, 21, 28, 29, 35, 54, 58, 59], respectively.

### 3.3. Research Trends Based on Research Methods

#### 3.3.1. Observational Study

*(1) Single Case Study*. Among the selected studies, single case studies were the most common (19 studies) [13–31]. Apart from 4 studies [13–16], all case studies were published after 2010; moreover, 10 and 9 studies were published in South Korea [14–17, 20–23, 27, 28] and China [13, 18, 19, 24–26, 29–31], respectively.

The study participants were 12 men and 7 women. The age of the participants ranged from 32 to 64 years; however, Lu and Zhao [26] did not mention the age of the participants. Two, seven, six, and three participants were in their 30s, 40s, 50s, and 60s, respectively, with a mean age of 49.22 years. Three, two, ten, three, and one participant developed ALS within 1 year, 1-2 years, 2–3 years, 3-4 years, and 6 years, respectively.

Qui et al. [29] used the El Escorial criteria for ALS diagnostic criteria; furthermore, 16 studies used electromyography (EMG) without specifying the diagnostic criteria [13–22, 24–28, 31]. Seven, two, and one study reported using magnetic resonance imaging (MRI) [14–17, 22, 26, 28], nerve conduction study (NCS) [14, 24], and genetic tests [20], respectively. Jeong et al. [21] conducted other various tests in addition to EMG; furthermore, Jo et al. [23] and Cao et al. [30] did not specify the diagnostic methods.

Four studies provided herbal medicine treatment as the only intervention [13, 25, 26, 30], while the remaining studies reported simultaneously using other treatments. Herbal medicines that were repeatedly used included Mazirensan [20, 21], Buzhongyiqitangjiajian [18, 24], Shiquandabutang [14, 23], Wujiapitang [20–22], Wugongtang [20–22], Wugongtangjiawei [21, 22], Dihuangyinzi [21, 22, 29], and Huangqiguizhiwuwutang [13, 19]. Combination treatments that were provided simultaneously with herbal medicines included riluzole [14–16, 29], trihexyphenidyl [20], acupuncture [15–17, 19–23, 27, 28, 31], pharmacopuncture [20–22, 28], burning acupuncture [17], electroacupuncture [17, 24], cupping therapy [17], moxibustion [18], Huangqi injection [18], exercise [15, 16], physical therapy [17, 23], manual therapy [23], and noninvasive BiPAP (bilevel positive airway pressure) ventilator [22]. The treatment duration differed across the studies, which ranged from 11 days to 12 years.

Clinical symptoms were assessed based on the ALS functional rating scale (ALSFRS) [14–17, 23, 27, 28], Medical Research Council (MRC) muscle scale [19, 28], Korean Oswestry Disability Index (K-ODI) [17], Simplified Nutritional Appetite Questionnaire score [21], global assessment scale [27], gait distance [27], weight [15, 21], body mass index [21], creatinine kinase level [28], saturation by pulse oximetry (SpO_2_) [22], end-tidal CO_2_ concentration [22], exhaled tidal volume [22], and clinical symptoms [13–20, 23–26, 29–31]. Clinical symptoms that improved after herbal medicine treatment included weakness, tongue numbness, gait ability, giddiness, lumbar pain, dysarthria, breathing, fatigue, stomach discomfort, constipation, mental state, loss of appetite, sweating, redness, muscle cramps, insomnia, dyspepsia, and sialorrhea. No studies reported on the safety and side effects.  Table 2 summarizes the details.

*(2) Case Series Studies*. Six case series studies were included [32–37]; among them, two, one, one, one, and one study were published in 1999 [32, 33], 2002 [34], 2003 [35], 2007 [36], and 2009 [37], respectively. Three and three studies were published in South Korea [35–37] and China [32–34], respectively.

These case series included 119 participants [32–37]. Excluding 18 participants included in the study by Kwon [35], which did not specify the sex, 60 and 41 of the remaining 101 participants were male and female, respectively. The mean age of the participants in the case series studies was 46 years, with the exclusion of participants in the studies by Cheng [33] and Kwon [35], which did not mention the mean age of the participants. The mean disease duration was 5, 1.67, and 4.75 years in the studies by Liang [32], Byun et al. [36], and Ryu et al. [37], respectively. In a study by Luo et al. [34], 2, 9, 8, and 7 of the 26 participants had ALS for a mean duration of >1 year, 1-2 years, 2-3 years, and 3-4 years, respectively. Cheng [33] and Kwon [35] did not mention the mean disease duration.

Luo et al. [34] used 3,200 diagnostic criteria for medical diseases to diagnose ALS. Three studies reported conducting EMG without mentioning the diagnostic criteria [32, 33, 35]; moreover, one study performed a biopsy examination to confirm ALS [35]. Byun et al. [36] and Ryu et al. [37] did not mention a diagnostic method.

None of the studies used herbal medicine as the only intervention, with all six studies providing herbal medicine treatment together with other treatments. Herbal medicine that were repeatedly used included Yangzuezhuangjinjianbuwan [35, 37], Banxiaxiexintang, Buzhongyiqitang, Bufeiyangyingjian, Qingzinreduotang, Qingzinlianzitang, Taorenchengqitang, Jiaweixiaoyaosan, Qiangjijianli oral liquid, Qiangjiling, Guzhenyinzi + Buzhongyiqitang, Guipitang + Wendantang, Xiangshapingweisan, Xiangshayangweitang, Erchentang, Erchentang + Siwutang, Renshenbaidusan, Renshenyangweitang, Zhengchuanjiaweierchentang, Zhenganxifengtangjiajian, Maqianzi powder, Bawutang, Sanqifuweiruansuowan, Xiaojinglongtang, Weizheng decoction, Yangyingjian, and Liujunzitang. Combination treatments that were provided with herbal medicine included riluzole [36, 37], acupuncture [32, 33, 35–37], pharmacopuncture [35], moxibustion [37], Huangqi injection [34], physical therapy (Qigong) [33], and Tuina therapy [34]. The treatment duration ranged from 1 to 24 months.

The assessed outcomes included the ALSFRS [35–37], ALS severity scale (ALSSS) [37], TER [32–34], and clinical symptoms [35, 37]. None of the studies reported safety and side effects.  Table 3 summarizes the details.

*(3) Retrospective Observational Study*. We included two retrospective observational studies [38, 39]; among them, one was published in 1997 [38] in Korea, while the other was published in 2017 in China [39]. The two studies included 305 participants (181 males and 124 females). In the study by Jeon et al. [38], the mean age of the participants was 52.4 years. Li et al. [39] did not report the mean age of the participants. None of the studies reported the mean disease duration. Li et al. used the El Escorial criteria for ALS diagnosis, while Jeon et al. [38] used the diagnostic criteria of Jokelainen et al.  Table 4 summarizes the details.

Jeon et al. [38] investigated the medical records of 17 hospitalized patients who were diagnosed with ALS; furthermore, they assessed the distribution of age and sex, ALS type, the period from onset to visiting an oriental hospital, hospitalization period, neurological signs, treatment method, and outcomes. Herbal medicine was the most common treatment method (17 (100%)), followed by acupuncture (16 (94.1%)), physical therapy (7 (41.2%)), moxibustion (5 (29.4%)), and cupping therapy (4 (23.5%)). Frequently prescribed herbal medicines included Buzhongyiqitang, Shiquandabutang, and Qingzaotang. Treatment outcomes included clinical symptoms such as muscle weakness, muscle atrophy, and speed disorder. These clinical symptoms were improved, unchanged, and aggravated in 4 (23.5%), 8 (47.1%), and 4 (23.5%) cases, respectively.

Li et al. [39] assessed the medical records of 288 hospitalized patients with ALS and analyzed the treatment methods, survival rate, and pattern identification types. In total, 152 (52.8%), 23 (8%), and 113 (39.2%) patients underwent herbal medicine treatment only, riluzole treatment only, and combination treatment with herbal medicine and riluzole, respectively. There was no significant among treatment difference in the survival period, as well as the 3- and 5-year survival rates. Analysis of the pattern identification types revealed 112 (39%) cases of spleen-stomach weakness pattern, 97 (34%) cases of spleen-kidney weakness pattern, 42 (15%) cases of liver-kidney yin deficiency pattern, 27 (9%) cases of phlegm-dampness pattern, 7 (2%) cases of static blood pattern, and 3 (1%) cases of dual deficiency of qi and yin pattern.

*(4) Prospective Observational Study*. We included one prospective observational study conducted by Pan et al. [8] in China that was published in 2013. Specifically, Pan et al. conducted a questionnaire on 231 patients with ALS (148 men and 83 women) who were treated at 12 hospitals in Shanghai to analyze the basic characteristics, current status, and reason for undergoing IT, use of herbal medicine, and integrated treatment efficacy and cost. The mean age of the participants and disease duration was 62.3 years and 2.1 years, respectively. The El Escorial criteria were used to diagnose ALS.  Table 5 summarizes the details.

Moreover, 299 (99%) participants reported using at least one integrative treatment. The main integrative treatment used included vitamin E (95%), coenzyme Q10 (95.47%), herbal extraction (94.8%), herbal decoction (90.6%), multivitamin (91%), and vitamin C (86.69%). The reasons for undergoing IT included weakness, fatigue, muscle atrophy, and delayed disease progression. The most commonly used herbal extracts included Jinkui Shenqi pills (Geumgwesingihwan in Korean; 13.09%), Buzhong Yiqi pills (Bojungikgihwan in Korean; 11.32%), Jianpi pills (Geonbihwan in Korean; 9.29%), Yangxue Qingnao granules (7.15%), Congrong Tongbian oral liquid (5.86%), and Baohe pills (Bohwahwan in Korean; 3.89%). Chinese herbal decoctions included Sijunzi decoction (Sagunjatang in Korean; 14.70%), Bazhen decoction (Paljintang in Korean; 12.34%), Shiquan Dabu decoction (Sibjeondaebotang in Korean; 8.31%), Buzhong Yiqi decoction (Bojung-iggitang in Korean; 6.68%), Tianwang Buxin Dan (Cheonwangbosimdan in Korean; 5.52%), and Guipi decoction (Gwibitang in Korean; 4.98%). Moreover, 63.23%, 24.69%, and 9.37% of the participants answered that integrative treatment had no significant effects, had mild effectiveness, and was effective, respectively. Treatment outcomes were mostly subjective improvement of clinical symptoms, including comfortable mood, happiness, vitality, sleep, increased appetite, and delayed disease progression.

#### 3.3.2. Intervention Studies

*(1) Before-and-after Study*. There were 12 before-and-after studies [40–51]; these studies have been consistently published from 2001 to 2019. Two studies [42, 43] and ten studies [40, 41, 44–51] were conducted in South Korea and China, respectively. Kim et al.'s study [43] was a follow-up study of a previous study [42]; moreover, studies by Li et al. [46] and Luo [47] were conducted using the same participants.

All 12 selected studies [40–51] were conducted prospectively without a control group; moreover, the researchers participated in the intervention. A total of 281 participants (192 men and 89 women) were included in the studies. Seven studies did not specify the mean age of the participants. The mean age of participants was 51.5, 50, 53.39, 55.65, and 55.9 years in the studies by Liu [40], Kim et al. [42], Kim et al. [43], Zhao [48], Wu [49], and Wen et al. [51], respectively. The average disease duration was 1.6 years in the study by Liu et al. [41], 18 months in the studies by Sun [44] and Zhao [48], 43.41 months in the studies by Kim et al. [42] and Kim et al. [43], 17.2 months in the study by Zhong [45], 18.74 months in the study by Wu [49], and 20 months in the studies by Li [46], Luo [47], and Wen et al. [51].

El Escorial criteria were used for ALS diagnosis in eight studies [42–49]. The remaining studies used the latest domestic and foreign disease diagnosis guidelines [40], the Chinese Medical Association Neurology Branch [41], and the Chinese guidelines for the diagnosis and treatment of ALS [50, 51]. These criteria were almost similar to the El Escorial criteria and had been translated into Chinese.

Seven studies described herbal medicine-only treatment. Liu et al. [41] administered combination therapy with Huagqi injection. Kim et al. [42] and Kim et al. [43] administered combination therapy with acupuncture, pharmacopuncture, and needle-embedding therapy. Meng et al. [50] administered acupuncture in combination, while Wen et al. [51] provided riluzole with herbal medicine treatment. Administered herbal medicines included Jianpibushen decoction [40], Jianpibushenxifeng decoction [41], Qiangjijianli capsule [41], Jianpiyifei decoction [44–48, 51], *Cervus elaphus* powder [50], and Qiangshenjianpi decoction [49]. Among them, Jianpiyifei decoction was the most commonly used (six studies) [44–48, 51]. In the studies by Kim et al. [42] and Kim et al. [43], pungent-dispersing and warm-relieving medicines for resolving phlegm, suppressing cough, detoxifying, and alleviating edema were mixed and used without a description being provided regarding the prescription name or its contents. Treatment was provided for as short as 8 to 9 months.

The assessed outcomes included the scores of the ALSFRS [42–51], ALSSS [45], MRC muscle scale [40, 42, 43], Norris [44], Appel [44], activities of daily living (ADL) Barthel index [50], Kubota drinking water test [45], Frenchay [45], dysphagia score [45], ALS assessment questionnaire 40 [46, 48], pulmonary function test [48], TER [40, 49, 51], average symptom score [40, 44, 47–51], EMG [40], and clinical symptoms [41]. Clinical symptoms that showed post-treatment improvement included speaking, eating, arranging the bed, running, and stair-climbing.

Most studies reported no side effects; moreover, three studies did not mention safety and side effects [41–43].  Table 6 presents the details.

*(2) Randomized Controlled Studies*. There were eight randomized controlled studies [52–59]; these studies have been published consistently from 2012 to 2020. Seven studies were conducted in China [52–58], and one study was conducted in Korea [59]. These studies included 306 participants (201 men and 105 women). The El Escorial criteria were used for ALS diagnosis in six studies [52, 54, 56–59]. Jin [53] used the ALS diagnostic criteria, while Fang [55] used the Chinese guidelines for the diagnosis and treatment of ALS, which is similar to the El Escorial criteria.

Four studies [54, 55, 58, 59] administered herbal medicine treatment as an intervention, while two studies [56, 57] combined herbal medicine treatment with general treatment. The other two studies [52, 53] administered acupuncture and conventional Western therapy to the experimental group. The herbal medicines used in these studies included Jiaweisijunzitang, Liqitongbianxieding decoction, Guiluerxianjiao, Jianpiyifei decoction, Jianpiliansetang, and Mecasin (also known as KCHO-1 or Gami-Jakyak Gamcho buja decoction). The control groups received riluzole or general treatment; moreover, four studies administered herbal medicine placebo [52, 57–59]. The treatment duration ranged from 14 days to 9 months.

Treatment outcomes included the scores of the ALSFRS [53–55, 57–59], ALSSS [55], short form-36 (SF-36) or short form-8 (SF-8) physical function [54, 59], Medical Research Council scale for muscle strength (MRC) [59], Epworth sleepiness scale [58], Hamilton rating scale for depression (HRSD) [59], fatigue severity scale (FSS) [59], visual analogue scale for pain (Pain VAS) [59], Kubota drinking water test [55], Frenchay [55], dysphagia score [55], bulbar symptoms self-score [55], mental fatigue score [56], pulmonary function test [52, 59], creatinine kinase [59], body weight [59], TER [52], average spleen deficiency symptom score [56], average TCM syndrome score [53, 57], patient global impression of change (PGIC) [59], and clinical symptoms [52, 56, 58].  Table 7 summarizes the details.

No side effects were reported in three studies [53, 57, 58]; moreover, two studies did not mention the side effects [55, 56]. Chin et al. [52] reported two cases of diarrhea in the experimental group, while Pan et al. [54] reported two cases of constipation and two cases of nausea in the experimental group. Yang [59] reported two cases of anxiety, two cases of cough, two cases of sputum, one case of insomnia, one case of hyperlipidemia, one case of shoulder pain, one case of low back pain, one case of tingling sense, one case of arm pain, one case of frequent voiding, and one case of upper respiratory infection in experimental group. The details are summarized in Table 7.

#### 3.3.3. Literature Studies

*(1) Nonsystematic Review*. We included seven nonsystematic review studies [7, 60–65], with one study each being published in 2007 [60], 2011 [61], 2015 [62], 2016 [63], 2017 [64], 2018 [65], and 2019 [7]. One study [7] and six studies [60–65] were conducted in South Korea and China, respectively.

Qiu [60] classified ALS into the liver-kidney yin deficiency pattern, spleen-stomach weakness pattern, and invading lung pattern. Huqianwanjiajian, Buzhongyiqitang + Shiquandabutang, and Baoyuanjian + Qingzaotangjiajian were mainly used for liver-kidney yin deficiency patterns, spleen-stomach weakness patterns, and invading lung patterns, respectively. Moreover, TCM has been reported as effective for improving symptoms such as shortness of breath, fatigue, sweating, heart burning, and lumbar pain in patients with ALS, as well as for extending the survival period and improving the quality of life of patients, which suggests the need for a well-designed study.

Zhao [61] classified ALS into the lung heat damaging fluid pattern, spleen-stomach weakness pattern, liver-kidney yin deficiency pattern, yin deficiency moving wind pattern, and spleen-kidney yang deficiency pattern. The most common treatments were Huqianwan, Dihuangyinzi, Bazhentang, Liuweidehuangwan, Zuoguiwan, Youguiwan, Dabuyinwan, and Tongqiaohuoxuetang. The authors classified and analyzed clinical studies on herbal medicine treatment for ALS based on the treatment method.

Xu et al. suggested that [62] Yiqiqiangjitang, Jianpibushenxifeng decoction, Zhiweitang, Qiangjinwan, Fuyuanshengji granule, Jiweiling extraction, and Shengjiqiangjinzhichantang can be used as herbal medicines for improving clinical symptoms, improving the quality of life, and reducing the financial burden on patients.

Deng [63] analyzed studies on medicines, medicine ingredients, prescriptions, and compounds used in cell and animal models of ALS, as well as humans. These studies used Huangqi, Fuyuanshengji granules, Jianpibushen decoctions, Bushenjianpishugan decoction, Guiluerxianjiao, Qiangjiling, Huangzitian, Jianpiyifeitang, Jiaweihuangqijianzhongtang + Huangqi injection, Jiaweijianbuhuqianwan, Qilongyiqisan, Wumeiwan, Maqianzi powder, and tonifying and replenishing medicinal prescriptions.

Mao [64] described findings regarding the effects of Sijunzitang, Yisuitang, Fuyuanshengji granule, Huqianwan, Jiweiling capsule, and Jiweiling injection on ALS. They suggested that compared with riluzole treatment, herbal medicine treatment had better outcomes in terms of cost and patients' quality of life.

Li and Zhan [65] described ALS as belonging to “wilting disease” and “impediment disease.” They classified ALS as dual deficiency of the lung-spleen pattern, dual deficiency of the spleen-kidney pattern, liver-kidney yin deficiency pattern, kidney yin, and yang deficiency pattern, and liver wind pattern based on scleral pattern identification. Furthermore, ALS can be classified into deficiency of original qi pattern, dual deficiency of qi and blood pattern, static blood pattern, and dampness obstruction pattern using qi-blood-phlegm-stasis pattern identification, as well as into disease of collateral vessels, governor vessels, and extra meridians using meridian pattern identification. The treatment used in the related studies included Qingjijianliyin; Qiangjijianli capsule; Qiangjijianli oral liquid; Shengyangyiweitang, Buzhongyiqitang, and Fujining capsule types 1 and 2; Buzhongyiqitang + Chaihushugansan; Mahuangfuzixixintang; Yiqijianpibuyuantang; Wenshenjianpishuangbu decoction; Zibuganshenyiqitang; Rencanhajietang; and Maqianzi powder.

Cai and Yang [7] analyzed studies on the CAM effects on ALS and reported that herbal medicine treatment improved disease symptoms. However, they indicated the need for large-scale clinical trials to allow the development of effective herbal medicine treatments. Related studies in this review used Buzhongyiqitang, Jiaweixiaoyaosan, Ziyinjianghuotang, Huolingshengji decoction, Dihuangyinzi, and Jiaweisijunzitang for prescription.

*(2) Systematic Reviews*. We included three systematic reviews [4, 66, 67]; among them, two studies were published in 2015 in Korea [66, 67] and one study was published in 2017 in China [4]. Kim et al. [66] reviewed 21 related studies to summarize the therapeutic effects of oriental medicine and TKM in patients with ALS. They observed that patients often underwent combined oriental treatment with herbal medicine as the main treatment; moreover, it was difficult to identify commonalities in selecting herbal medicine prescriptions. None of the studies reported the cure for ALS. However, combined oriental treatment was found to improve the main ALS symptoms, including muscle weakness, dysphagia, dysarthria, and respiratory muscle weakness, which leads to long-term management.

Lee et al. [67] reviewed 18 studies, including retrospective observational studies, prospective clinical studies, and case reports, on oriental medicine treatment for ALS in South Korea. It was found that IT using herbal medicine, acupuncture, and herbal acupuncture was the most commonly used; moreover, there were cases with short-term positive improvements in the main ALS symptoms.

Zhu et al. [4] analyzed the literature to summarize the characteristics of TCM and other drugs used for ALS prevention and treatment. However, they did not mention the number of studies reviewed. They found that tonifying kidney, tonifying lung, tonifying qi, and modulating meridian can be performed for treatment; moreover, TCM has several benefits, including low treatment cost, minor side effects, longevity, and improved quality of life.

*(3) Meta-Analysis*. Only Li et al. conducted a meta-analysis after a systematic review of the literature [68]. This study was conducted in China and published in 2016. They assessed the efficacy and safety of tonifying and replenishing medicinal prescriptions for ALS. Seven randomized controlled trials were selected that analyzed 411 cases (257 and 154 cases in the treatment and control groups, respectively). The El Escorial criteria were used as the diagnostic criteria. The treatment group received tonifying and replenishing medicinal prescriptions, including Buyitangjiajian, Fuyuanshengji granule, Shengjiqiangjinzhichantang, Jiaweisijunzitang, and Yiqiqiangjitang. On the other hand, the control group received riluzole except in one study. TER was significantly higher in the treatment group than in the control group; furthermore, the decrease in the Norris score was significantly lower in the treatment group than in the control group. Additionally, the decrease in the average TCM syndrome score was significantly higher in the treatment group than in the control group. Therefore, compared with riluzole, TCM was more effective in delaying disease progression without significantly improving or treating the symptoms. However, TCM is cheaper than riluzole and can be conveniently stored, which reduces the economic burden on patients. There were no reports of serious safety-related adverse events.

## 4. Discussion

We conducted a scoping review on Korean and other international studies to understand the research trends in ALS herbal medicine treatment. Regarding the year and format of the published studies, studies on herbal medicine treatment for ALS have been published since 1995. Moreover, approximately 70% of the studies were published from 2010 to 2019, which indicates that studies have been more actively conducted in recent years. The included studies were conducted in China and South Korea, with over 70% of the studies being conducted in China. Accordingly, most of the studies were published in Chinese (over 60%). Among the 59 selected studies, a majority were observational studies (47.5%), followed by intervention studies (33.9%) and literature studies (18.6%). Further classification revealed that case reports (single case reports and case series) and before-and-after studies accounted for 42.4% and 20.3% of all the studies, respectively. Randomized controlled studies and narrative review studies accounted for 13.6% and 11.9% of the included studies. Among the included studies, randomized controlled trials, systematic reviews, and meta-analyses have been published since 2012; moreover, only one meta-analysis, which presented the highest level of evidence, was published in China by Li et al. in 2016 [68]. To select relevant studies, we searched for herbal medicines used in South Korea, China, and Japan; accordingly, studies on other herbal products and single extracts from other countries were excluded. Consequently, the selected studies were limited to East Asia. The ALS prevalence in South Korea is low; therefore, it is difficult to recruit participants in South Korea, which led to the inclusion of a significantly smaller number of clinical studies conducted in South Korea compared with those conducted in China. There is a need for a review that includes a greater scope of interventions to assess global research trends.

ALS is mostly diagnosed based on symptoms of progression of the upper and lower motor nerves, which are mainly assessed through medical history and neurological examinations. Furthermore, it is important to conduct EMG, nerve conduction velocity tests, laboratory tests, and imaging tests to exclude other possible nervous system disorders with similar symptoms, including peripheral neuropathy, muscle disease, neuromuscular junction disease, and central nervous system disease [1]. The most commonly used diagnostic criteria are the El Escorial criteria revised in 2000 (Table 8). These diagnostic criteria are based on identifying lower motor neuron abnormalities through clinical symptoms and EMG, assessing upper motor neurons through clinical manifestations [69], and evaluating the invasion level in the soft, cervical, thoracic, and lumbar plexus [69]. Among the 59 selected studies, only 19 studies (approximately 32%) mentioned the El Escorial criteria [8, 29, 39, 42–49, 52, 54, 56–59, 66, 68]. In China, there are diagnostic criteria similar to the El Escorial criteria, including the Chinese Medical Association Neurology Branch [41], Guidelines for the diagnosis and treatment of ALS in China [50, 51, 55], and the latest domestic and foreign disease diagnosis guidelines [40]. Most of the included case reports mentioned that EMG, nerve conduction velocity tests, and magnetic resonance imaging tests were performed without using the diagnostic criteria. ALS is often misdiagnosed due to the low ALS incidence rate, atypical clinical symptoms in the early stage of onset, and low awareness among doctors of specialties other than neurologists [39]. Therefore, there is a need to explain the diagnosis process of the disease based on the diagnostic criteria in case reports to provide clear evidence regarding the diagnosis.

In the selected studies, ALS was diagnosed as a “wilting disease.” Additionally, prescriptions with anti-inflammatory, protein aggregation inhibition, and anti-oxidant properties, including Buzhongyiqitang, Jiaweisijunzitang, Qiangjijianli decoction, Jianpiyifei decoction, Dihuangyinzi, Bawutang, Huqianwan, Fuyuanshengji granule, and Mecasin were mostly used [4, 59]. In China, ALS is thought to involve the liver, spleen, lungs, and kidneys; moreover, different treatments are provided based on the ALS type. Contrastingly, in South Korea, treatment was selected on an individual basis to treat the main and secondary clinical symptoms. Therefore, it was difficult to determine common points in the herbal medicine prescription between South Korean and Chinese studies; moreover, we could not determine the association between specific prescriptions and outcome improvement. Therefore, future studies should investigate the prescription types based on comparisons between the characteristics of TCM and TKM.

The selected studies used various evaluation tools to assess ALS progression. The most representative tool was ALSFRS, which is a motor ability evaluation index for patients with ALS that was developed by Brooks. It comprises 10 items evaluated on a total of 40 points with a maximum of four points for each item. This tool assesses the physical functions required for daily life. Moreover, it has been supplemented with detailed items regarding respiratory function in the ALSFRS-Revised (ALSFRS-R) [70]; furthermore, the K-ALSFRS-R was developed for use in South Korea with translation and modification of the tool to suit the characteristics of South Korean patients [71]. The ALSFRS-R is relatively easy to use since it allows assessment of the patient via a phone call through a guardian [72]. Among the selected studies, 28 (47.5%) studies used the ALSFRS [14–17, 23, 27, 28, 35–37, 42–51, 53–55, 57–59, 66, 67]; furthermore, the ALSFRS score was significantly increased in three studies [17, 27, 50]. Yeon et al. [17] reported that acupuncture; burning acupuncture; electroacupuncture; cupping therapy; and physical therapy involving herbal medicine with dispelling wind-dampness, relieving meridian, and sinew- and bone-strengthening properties improved lumbar pain, K-ODI scores, and K-ALSFRS-R scores. Kim et al. [27] reported an improvement in the GAS scores, K-ALSFRS-R scores, and gait distance after administration of Jianghuodihuangtang and Wujiapizhuangjitang, followed by acupuncture and Saam acupuncture for 11 days, in a 52-year-old female patient. Moreover, Meng [50] reported an improvement in the scores of the ALSFRS-R and ADL-Barthel index after the prescription of *Cervi parvum* cornu extraction and acupuncture in 29 patients with ALS. Sixteen studies [15, 16, 28, 35, 36, 42–44, 46–49, 51, 55, 58, 59] reported no or nonsignificant changes in the ALSFRS scores. Jin [53], Pan et al. [54], Zhu et al. [57], and Yang [59] reported a decrease in the ALSFRS score; however, the decrease was significantly less than that in the control group. This suggests that compared with the control group, the treatment group showed slower disease progression. However, this study did not consider the disease duration, which decreased the objectivity of the findings. The progression rate of ALS (ΔFS) is a quantitative index used for assessing disease progression in patients. This was calculated by determining the difference between the ALSFRS-R scores at the evaluation time and symptom onset, followed by dividing it by the number of months from the time of symptom onset to the evaluation time. This yields the progression speed and prognosis of a disease, which allows objective assessment of the treatment effects [72, 73]. No study on herbal medicine has used ΔFS, which may be useful for future studies.

Among the selected studies, only two reported cases of ALS being cured [13, 25]. Other studies reported delayed disease progression after herbal medicine treatment or combined herbal treatment, as well as improvements in appetite loss, drooling, muscle spasms, fatigue, shortness of breath, constipation, gait, and sweating. However, there remains no objective evaluation tool for assessing clinical symptoms. Therefore, patient symptoms were subjectively evaluated. Future studies should employ objective tools for assessing the clinical symptoms of patients.

Adverse events were reported in three randomized controlled studies. Chin et al. [52] reported two cases of diarrhea in the experimental group, while Pan et al. [54] reported two cases of diarrhea and nausea in the experimental group. Yang et al. [59] reported two cases of anxiety, two cases of cough, two cases of sputum, one case of insomnia, one case of hyperlipidemia, one case of shoulder pain, one case of low back pain, one case of tingling sense, one case of arm pain, one case of frequent voiding, and one case of upper respiratory infection in the experimental group. Although most studies reported no side effects, some studies did not mention side effects at all. Therefore, future studies should further assess the side effects of herbal medicine treatment.

A study conducted by the German Association for Neuromuscular Diseases [10] reported that 54% of patients with ALS had received CAM; among them, 60% had experienced positive effects. The reasons for using CAM included improving general health, delaying disease progression, improving muscle strength, and treating the disease [10]. A Chinese study reported that 99% of patients with ALS had received IT, while 94.8% and 90.6% had used herbal extraction and decoction, respectively. The reasons for using IT included weakness, fatigue, muscle atrophy improvement, and delayed disease progression. However, only 9.37% of the participants answered that the treatment was effective with most of them considering IT as ineffective [8]. In a study conducted in South Korea, all patients used CAM with the expectation of improvement in symptoms. However, 70.6% of the patients were not satisfied with the outcome and discontinued CAM; furthermore, only 14.5% reported intending to recommend CAM to others [9]. ALS progression is rapid and fatal. However, there remains no fundamental cure for ALS; moreover, treatment is difficult in many cases. Consequently, there is increased interest in CAM and integrative medicine among patients with ALS. However, no studies have investigated patients' perceptions of and demand for CAM [74]. Therefore, there is a need for future studies on CAM and IT to provide better herbal medicine treatment to patients.

This current systematic scoping review analyzed studies in electronic academic databases to understand the research trend of herbal medicine treatment for ALS. ALS shows rapid progression with a high mortality rate, which makes it difficult to conduct randomized controlled studies. Specifically, there are few randomized controlled studies and meta-analysis studies on ALS in East Asia. In the selected case report studies, a detailed assessment of the effects of herbal medicine treatment alone could not be achieved since they administered herbal medicine together with acupuncture, pharmacopuncture, cupping therapy, or physical therapy.

The present scoping review has the strength of being the first study to systematically search the electronic database and synthesize the results of the latest studies related to East Asian herbal medicine published up to 2021. In this review, not only representative domestic databases of China, Korea, and Japan but also international databases such as PubMed, Embase, Scopus, and Cochrane have been used to collect published studies. The previously published narrative reviews did not use a systematic search method [7, 60–65], and systematic reviews also had limitations in that they contained content limited to one of the East Asian countries such as China [4] or Korea [66, 67]. Furthermore, the meta-analysis published in 2016 [68] did not include relatively high-quality randomized controlled trials published after 2017 [57–59].

Among the selected 59 studies, we focused on two recent randomized controlled studies published by Zhu et al. [57] and Yang [59]. According to our search results, randomized controlled trials evaluating the clinical effects of traditional East Asian herbal medicine on ALS have been published since 2012 [52]. In both studies, higher-level research methodologies were used compared to the existing randomized controlled trials [52–56]. Both studies used placebo as a control treatment, and ALSFRS-R scores, one of the most standard evaluation tools, were used to evaluate the effects of interventions on the progression of ALS. In each study, Jiaweisijunzitang and Mecasin were used, respectively. As a result, it was confirmed that the progression of ALS was slowed after 9 months and 12 weeks, respectively, compared to the control group. Although the level of evidence is not yet high, as mentioned above, Jiaweisijunzitang and Mecasin are also noteworthy as future therapeutic agents for ALS, as they also have mechanisms of pharmacological effects such as anti-inflammatory, protein aggregation inhibition, and anti-oxidant effect [4, 59]. Given that there remains no fundamental cure for ALS, CAM is commonly used for ALS treatment. Among CAM, herbal medicine treatment can improve the immune response, slow disease progression, and improve symptoms through anti-inflammatory, anti-oxidant, and protein-aggregation inhibition effects. This study is significant since it highlights the shortcomings of existing studies to suggest possible directions and aims for future studies. Future studies with detailed designs could yield evidence regarding the effects of herbal medicine treatment for ALS and develop better treatments for patients with ALS.

## 5. Conclusion

This scoping review revealed that there remained insufficient data on the effects of herbal medicine treatment for ALS even though there has been active research in recent years. To provide effective herbal medicine treatment to patients with ALS, systematic and elaborately designed high-quality clinical studies are required to demonstrate the benefits of herbal medicine treatment.

## Figures and Tables

**Table 1:** General characteristics of the included literature (*N* = 59).

Variables	Categories	*n* (%)
Publication year	1995–1999	4 (6.8)
	2000–2004	7 (11.9)
	2005–2009	6 (10.2)
	2010–2014	19 (32.2)
	2015–2019	23 (40.0)
Research methodology	Observational studies	28 (47.5)
	Case report	19 (32.2)
	Case series	6 (10.2)
	Retrospective study	2 (3.4)
	Prospective study	1 (1.7)
	Experimental studies	20 (33.9)
	Before-and-after study	12 (20.3)
	Randomized controlled trial	8 (13.6)
	Literature studies	11 (18.6)
	Narrative review	7 (11.9)
	Systemic review	3 (5.1)
	Meta-analysis	1 (1.7)
Country	China	39 (66.1)
	Korea	20 (33.9)
Language	Chinese	34 (57.6)
	Korean	14 (23.7)
	English	11 (18.6)

**Table 2:** Review of case reports.

First author (year)	Country	Sex	Age	Duration of disease	Diagnosed by	Herbal medicine	Treatment duration	Other intervention	Outcomes	Significant finding
Wu (1995) [13]	China	M	37	2 y	EMG	Maqianzi powder Huangqiguizhiwuwutang Siwutang	No mention	No mention	Clinical symptoms	Improving muscle strength No recurrence after cure
Kim et al. (2000) [14]	Korea	F	40	8 m	EMG NCS MRI	Shiquandabutang Buzhongyiqitang Reduohanshaotang Bawutang Bawutangjiawei	8 m	Riluzole	Clinical symptoms ALSFRS	Reducing dependence on a ventilator
Park et al. (2001) [15]	Korea	F	60	6 m	EMG MRI	Shaoyaogancaotangjiawei Sijunzi + lizhongtangjiawei	2 m	Acupuncture Exercise Riluzole	Clinical symptoms ALSFRS Body weight	Improving dysphagia, lalopathy, and hypotonia
Kim et al. (2004) [16]	Korea	F	50	8 m	EMG MRI	Jiaweixiaoyaosan Ziyindabuwanjiawei	1 m	Riluzole Acupuncture Exercise	Clinical symptoms ALSFRS	Improving facial flushing and sweating
Yeon et al. (2010) [17]	Korea	M	32	2 y	EMG MRI	Combination of several herbal medicines^*∗*^	1 m	Acupuncture Burning Acupuncture Electroacupuncture Cupping therapy Physical therapy	Clinical symptoms K-ODI K-ALSFRS-R	Improving low back pain, K-ODI scores, and K-ALSFRS-R scores
Wang et al. (2010) [18]	China	M	54	2 y	EMG	Buzhongyiqitangjiajian Qiangjijianli oral liquid	8 m	Huangqi injection Moxibustion	Clinical symptoms	Improving dysphagia, muscle strength, and appetite
Liang et al. (2012) [19]	China	F	48	3 y	EMG	Huangqiguizhiwuwutang	3 m	Acupuncture	Clinical symptoms MRC muscle scale	Improving gait, dizziness, and muscle strength
Jeong et al. (2013) [20]	Korea	M	61	2 y	EMG Genetic testing	Wugongtang Wujiapitang Mazirensan	3 m	Trihexyphenidyl (stopped) Acupuncture pharmacopuncture	Clinical symptoms	Decreasing salivation
Jeong et al. (2013) [21]	Korea	M	53	2 y 3 m	EMG Several tests	Dihuangyinzi Wugongtang Wugongtangjiawei Wujiapitang Mazirensan	9m	Acupuncture Pharmacopuncture	Body weight BMI SNAQ score	Increasing body weight, BMI, and SNAQ score
Lee et al. (2014) [22]	Korea	M	56	2 y	EMG MRI	Chungpajeongami Dihuangyinzi Wugongtang Wugongtangjiawei Wujiapitang Dansengcangzhutang Shaoyaogancaotang Dansengcangzhutianmagancaotang	3 y	Acupuncture Scolopendrid pharmacopuncture Bee-venom pharmacopuncture Noninvasive BIPAP ventilator	SpO_2_ EtCO_2_ Vte	Maintaining SpO_2_, EtCO_2_, and Vte
Jo et al. (2014) [23]	Korea	M	49	20 m	No mention	Shiquandabutang	5 m	Acupuncture Physical therapy Manual therapy	Clinical symptoms K-ALSFRS-R	Maintaining respiratory function and speech
Sun and Xu (2014) [24]	China	M	42	2 y	EMG NCS	Buzhongyiqitangjiajian	2 m	Electroacupuncture	Clinical symptoms	Reducing fatigue, stomach discomfort, and constipation Improving muscle strength and mental state Gaining weight
Zhong et al. (2014) [25]	China	M	57	1 y	EMG	Combination of several herbal medicines^*∗*^	1 y	No mention	Clinical symptoms	Improving muscle strength
Lu and Zhao (2015) [26]	China	F	No mention	1 y	EMG MRI	Bushenkangshuaipian Combination of several herbal medicines^*∗∗*^	3 w	No mention	Clinical symptoms	Improving mental state, appetite, and muscle strength Reducing fatigue
Kim et al. (2016) [27]	Korea	F	52	6 y	EMG	Jianghuodihuangtangjiajian Wujiapizhuangjitangjiawei	11 d	Acupuncture	GAS Walking distance K-ALSFRS-R	Improving the GAS of the weakness of the upper and lower extremities Increasing walking distance
Cha et al. (2016) [28]	Korea	M	43	3 y	EMG MRI	Glycyrrhiza uralensis extract Jiaweishaoyaogancaofuzitang	3 m	Acupuncture Pharmacopuncture	CK level ALSFRS-R MRC muscle scale	Decreasing CK levels No change in the score of the MRC muscle scale
Qiu et al. (2016) [29]	China	F	41	3 y	El Escorial criteria	Dihuangyinzi	12 y	Riluzole (stopped after 1 month)	Clinical symptoms	Not requiring permanent continuous ventilator Improving choking on liquids
Cao et al. (2017) [30]	China	M	47	2 y	No mention	Liuweidihuangwan + Buzhongyiqitangjiajian	6 w	No mention	Clinical symptoms	Reducing sweating, hot flash, muscle fibrillation, improving muscle strength, appetite, and sleep
Liu et al. (2018) [31]	China	M	64	2 y	EMG	Liuweidihuangtang + Bazhentangjiajian	2 m	Acupuncture	Clinical symptoms	Improving weakness of the upper limbs and tongue stiffness

^*∗*^Details are not specified. ^*∗∗*^Astragali Radix, Codonopsis Pilosulae Radix, Achyranthis Radix, Eucommiae Cortex, Homalomenae Rhizoma, Cnidii Rhizoma, Psoraleae Semen, Salviae Miltiorrhizae Radix, Leonuri Herba, Glycyrrhizae Radix et Rhizoma, and Ostreae Testa. M, male; F, female; d, day(s); w, week(s); m, month(s); y, year(s); EMG, electromyography; MRI, magnetic resonance imaging; NCS, nerve conduction study; ALSFRS, amyotrophic lateral sclerosis functional rating scale; ALSFRS-R, ALSFRS-Revised; K-ALSFRS-R, Korean-ALSFRS-R; K-ODI, Korean Oswestry Disability Index; MRC, Medical Research Council; SNAQ, Simplified Nutritional Appetite Questionnaire; GAS, global assessment scale; BMI, body mass index; CK, creatine kinase; SpO2, saturation by pulse oximetry; EtCo2, =end-tidal CO_2_ concentration; and Vte, exhaled tidal volume.

**Table 3:** Review of the case series.

First author (year)	Country	Sample size	Sex (M: F)	Average age	Duration of disease	Diagnosed by	Herbal medicine	Duration of treatment	Other intervention	Outcomes	Significant finding
Liang (1999) [32]	China	24	17:7	43	5 y	EMG	Maqianzi powder Combination of several herbal medicines^*∗*^	No mention	Acupuncture	TER	TER 91.67%
Cheng Y (1999) [33]	China	46	27:19	No mention	No mention	EMG	Sanqifuweiruansuowan	6–24 m	Acupuncture Physical therapy(Qigong)	TER	TER 89.13%
Luo et al. (2002) [34]	China	26	15:11	47.9	No mention	3,200 diagnostic criteria for medical diseases	Qiangjiling Qiangjijianli oral liquid	3 m	Huangqi injection Tuina therapy	TER	TER 68.75%
Kwon (2003) [35]	Korea	18	No mention	No mention	No mention	EMG biopsy	Yangzuezhuangjinjianbuwan	3–6 m	Acupuncture Pharmacoacupuncture Bee-venom pharmacoacupuncture	Clinical symptoms ALSFRS	Inhibition of progression Improving local symptoms Psychological stability
Byun et al. (2007) [36]	Korea	3	0:3	53.3	1.67 y	No mention	Liujunzitang Weizheng decoction Yangyingjian Guzhenyinzi + Buzhongyiqitang Erchentang Bufeiyangyingjian Buzhongyiqitang Jiaweixiaoyaosan Xiangshayangweitang Zhengchuanjiaweierchentang Renshenyangweitang Erchentang + Siwutang Guipitang + Wendantang Renshenbaidusan	1.5–6 m	Acupuncture riluzole	ALSFRS	Delayed symptoms
Ryu et al. (2009) [37]	Korea	2	1:1	50	4.75 y	No mention	Bawutang Xiangshapingweisan Xiaojinglongtang Taorenchengqitang Banxiaxiexintang Qingzinlianzitang Qingzinreduotang Yangzuezhuangjinjianbuwan Zhenganxifengtangjiajian	1–2 m	Acupuncture Moxibustion riluzole	K-ALSFRS-R ALSSS Clinical symptoms	Improving local symptoms

^*∗*^Astragali Radix, Codonopsis Pilosulae Radix, Atractylodis Rhizoma Alba, Citri Unshius Pericarpium, Gastrodiae Rhizoma, Bupleuri Radix, Rehmanniae Radix Preparata, Rehmanniae Radix Recens, Dipsaci Radix, Cuscutae Semen, Lycii Fructus, Epimedii Herba, Phellodendri Cortex, Anemarrhenae Rhizoma, Paeoniae Radix, and Glycyrrhizae Radix et Rhizoma. M, male; F, female; m, month(s); y, year(s); TER, total effective rate; ALSFRS, amyotrophic lateral sclerosis functional rating scale; K-ALSFRS-R, Korean-ALSFRS-Revised; and ALSSS, Amyotrophic lateral sclerosis severity scale.

**Table 4:** Review of retrospective studies.

First author (year)	Country	Sample size	Sex (M:F)	Average age	Duration of disease (average)	Diagnosed by	Outcomes
Jeon et al. (1997) [38]	Korea	17	12:5	52.4	No mention	Diagnostic criteria by Jokelainen et al.	Analyzing the age of onset, clinical signs, ALS type, treatment methods, outcomes, etc.
Li et al. (2017) [39]	China	288	169:119	No mention	No mention	El Escorial criteria	Analyzing the clinical characteristics, survival rate, and TCM syndrome in patients with ALS

M, male; F, female; ALS, amyotrophic lateral sclerosis; and TCM, traditional Chinese medicine.

**Table 5:** Review of prospective Study.

First author (year)	Country	Sample size	Sex (M:F)	Average age	Duration of disease (average)	Diagnosed by	Outcomes
Pan et al. (2013) [8]	China	231	148:83	63.2	2.1 y	El Escorial criteria	Analyzing the basic patient characteristics, IT usage status, reasons for using IT, Chinese medicine usage status, IT efficacy, and IT cost

M, male; F, female; y, year(s); and IT, integrative therapy.

**Table 6:** Review of before-and-after studies.

First author (year)	Country	Sample size	Sex (M:F)	Average age	Duration of disease (average)	Diagnosed by	Herbal medicine	Duration of treatment	Other intervention	Outcomes	Significant findings	Side effects
Liu (2001) [40]	China	32	24:8	51.5	No mention	The latest domestic and foreign disease diagnosis guidelines	Jianpibushen decoction	9 m	None	TER TCM syndrome score MRC muscle scale EMG	TER 84.38% Improvement in tongue quality and pulse Increasing the motor neuron conduction rate Reduction in the possibility of fascia fibrillation	None
Liu et al. (2006) [41]	China	40	31:9	No mention	3–36 m (19.2 m)	Chinese Medical Association Neurology Branch	Jianpibushenxifeng decoction Qiangjijianli capsule	3 m	Huangqi injection	Clinical symptoms	Improvement in speaking, eating, bed arrangement, running, and climbing stairs	No mention
Kim et al. (2009) [42]	Korea	12	6:6	50	(43.41 m)	El Escorial criteria	Combination of several herbal medicines^*∗*^	30 d	Acupuncture Bee-venom pharmacoacupuncture Scolopendrid pharmacopuncture Fel Ursi pharmacoacupuncture Needle-embedding therapy	ALSFRS-R MRC muscle scale	An increase in the scores of the ALSFRS-R and MRC muscle scale	No mention
Kim et al. (2010) [43]								3 m	Acupuncture Bee-venom pharmacoacupuncture Scolopendrid pharmacopuncture Saline injection Needle-embedding therapy	ALSFRS-R MRC muscle scale	Slower progression compared with patients without Oriental medical treatment	No mention
Sun (2009) [44]	China	23	18:5	No mention	3–84 m (18 m)	El Escorial criteria	Jianpiyifei decoction	3 m	None	ALSFRS Norris Appel TCM syndrome score	Enhancement of motor functions(feeding, clothing, writing, and bed emancipated) Improvement in hypodynamia, palpitation, sweating, appetite, and pale tongue	None
Zhong (2011) [45]	China	20	14:6	No mention	2–72 m (17.2 m)	El Escorial criteria	Jianpiyifei decoction	5 m	None	ALSFRS-R ALSSS kubota drinking water test Frenchay Dysphagia score	Delayed disease progression	None
Li (2012) [46]	China	28	17:11	No mention	3–64 m (20 m)	El Escorial criteria	Jianpiyifei decoction	2 m	None	ALSFRS-R ALSAQ-40	Stable clinical symptoms Improvement of quality of life	None
Luo (2012) [47]										TCM syndrome score ALSFRS	Delayed disease progression	None
Zhao (2013) [48]	China	36	23:13	53.39	3–64 m (18 m)	El Escorial criteria	Jianpiyifei decoction	2 m	None	MRC muscle scale ALSFRS-R ALSAQ-40 TCM syndrome score Lung function scale	Maintenance of quality of life, motor function, and lung function	None
Wu (2015) [49]	China	31	22:9	55.65	1–62 m (18.74 m)	El Escorial criteria	Qiangshenjianpi decoction	3 m	None	ALSFRS-R TCM syndrome score TER	Improvement of the TCM symptom score TER 87.10%	None
Meng (2018) [50]	China	29	19:10	No mention	No mention	Guidelines for the diagnosis and treatment of ALS in China	*Cervus elaphus* powder	3 m	Acupuncture	TCM syndrome score ADL-Barthel index ALSFRS-R	Improvement of the TCM syndrome score, ADL-Barthel index, and ALSFRS-R	None
Wen et al. (2019) [51]	China	30	18:12	55.9	3–84 m (20 m)	Guidelines for the diagnosis and treatment of ALS in China	Jianpiyifei decoction	8 w	Riluzole	ALSFRS-R TCM syndrome score TER	Improvement of the TCM symptom score TER 56.7%	None

^*∗*^Details are not specified. M, male; F, female; d, day(s); w, week(s); m, month(s); TER, total effective rate; TCM, traditional Chinese medicine; MRC, Medical Research Council; EMG, electromyography; ALSFRS, amyotrophic lateral sclerosis functional rating scale; ALSFRS-R, ALSFRS-Revised; ALSSS, Amyotrophic lateral sclerosis severity scale; ALSAQ, Amyotrophic lateral sclerosis assessment questionnaire; and ADL, activities of daily living.

**Table 7:** Review of randomized controlled trials.

First author (year)	Country	Sample size (T/C)	Sex (M:F)	Average age (T/C)	The average duration of disease (T/C) (m)	Diagnosed by	Intervention	Duration of treatment	Outcomes	Significant findings	Side effects (*n*)
Chin et al. (2012) [52]	China	37 (23/14)	(T) 18:5 (C) 10:4	74.5	No mention	El Escorial criteria	(T) Liqitongbianxieding decoction, general treatment, acupuncture, massage (C) Placebo, general treatment	14d	TER Number of evacuation Stool characteristics Symptoms of difficulty in evacuation Companion symptoms during the evacuation Pulmonary function test	TER T 100%, C 83.3% Improving number of evacuation, stool characteristics, symptoms of difficulty in evacuation, and companion symptoms during evacuation Improving peak airway pressure, respiratory frequency, and airway resistance	Diarrhea(2)
Jin (2013) [53]	China	28 (15/13)	(T) 11:4 (C) 9:4	54.45 (57.83/50.54)	(18.62/18.25)	ALS diagnosis criteria	(T) Guiluerxianjiaojiajian, riluzole, acupuncture (C) Riluzole	6 m	ALSFRS-R TCM syndrome score	Compared with the control group, the treatment group showed a lower decrease in the ALSFRS-R score	None
Pan et al. (2013) [54]	China	42 (23/19)	(T) 14:9 (C) 11:8	50.4 (51.6/50.1)	(25.9/26.1)	El Escorial criteria	(T) Jiaweisijunzitang (C) Riluzole	6 m	ALSFRS SF-36 physical function	Limbs first attacked in patients in the treated group had a smaller rate of change of ALSFRS scores	Constipation(2) Nausea(2)
Fang (2016) [55]	China	37 (18/19)	(T) 13:5 (C) 11:8	51.31 (49.1/53.4)	(17.6/12.9)	Guidelines for the diagnosis and treatment of ALS in China	(T) Jianpiyifei decoction (C) Riluzole	12w	Frenchay Dysphagia score kubota drinking water test speech-ALSSS swallowing-ALSSS ALSFRS-R bulbar symptoms self-score	No statistical significant difference	No mention
Bao et al. (2016) [56]	China	45 (24/21)	(T) 15:9 (C) 14:7	No mention	No mention	El Escorial criteria	(T) Jiaweisijunzitang, general treatment (C) General treatment	6 m	TCM spleen deficiency symptoms score Shortness of breath Mental fatigue score	Delayed TCM deterioration Spleen deficiency symptoms, Shortness of breath, and mental fatigue	No mention
Zhu et al. (2017) [57]	China	45 (24/21)	(T) 15:9 (C) 14:7	No mention	No mention	El Escorial criteria	(T) Jiaweisijunzitang, general treatment (C) Placebo, general treatment	9 m	ALSFRS-R TCM syndrome score	Delaying the deterioration of related symptoms and ALSFRS-R	None
Gao et al. (2017) [58]	China	42 (22/20)	(T) 14:8 (C) 13:7	50.4 (50.2/50.7)	(23.9/24.3)	El Escorial criteria	(T) Jian Pi Lian Se Tang (C) Placebo, general treatment	6 w	QS FNU ESS ALSFRS	Improving the scores of the QS, FNU, and ESS	None
Yang (2019) [59]	Korea	30 (19/11)	(T-1) 5:5 (T-2) 7:3 (C) 7:3	56.47 (54.50/58.30/56.60)	No mention	El Escorial criteria	(T-1) Mecasin^*∗*^ 1.6 g, riluzole (T-2) Mecasin^*∗*^ 2.4 g, riluzole (C) Placebo, riluzole	12 w	ALSFRS-R SF-8 MRC scale for muscle strength VAS pain HRSD FSS PGIC Pulmonary function test Creatinine kinase Body weight	K-ALSFRS-R were significantly decreased in the (C) compared to (T-1), (T-2)	(T) Anxiety (2), Cough (2), Sputum (2), Insomnia (1), Hyperlipidemia (1), Shoulder pain (1), Low back pain (1), Tingling sense (1), Arm pain (1), Frequent voiding (1), Upper respiratory infection (1) (C) Insomnia (2), Constipation (1),

^*∗*^ *Curcuma longa*, *Salvia miltiorrhiza*, *Gastrodia elata*, *Chaenomeles sinensis*, *Polygala tenuifolia*, *Paeonia lactiflora*, *Glycyrrhiza uralensis*, *Atractylodes lancea*, and *Aconitum carmichaeli*. It is a 30% ethanol extraction. T, treatment group; C, control group; M, male; F, female; w, week(s); m, month(s); TER, total effective rate; ALSFRS, amyotrophic lateral sclerosis functional rating scale; ALSFRS-R, ALSFRS-Revised; SF, short from; TCM, traditional Chinese medicine; ALSSS, amyotrophic lateral sclerosis severity scale; ESS, Epworth sleepiness scale; QS, quantity of salivation/sialorrhea; FNU, frequent nighttime urination; MRC, Medical Research Council; VAS, visual analogue scale; HRSD, Hamilton rating scale for depression; FSS, fatigue severity scale; and PGIC, patient global impression of change.

**Table 8:** Summary of the revised El Escorial Diagnostic criteria for ALS.

Diagnosis	Involved segments
Clinically possible ALS	UMN and LMN signs in one region; UMN signs in at least two regions; or UMN and LMN signs in two regions without UMN signs rostral to the LMN signs
Laboratory-supported probable ALS	UMN signs in one or more regions and LMN signs in at least two regions defined by electromyography
Clinically probable ALS	UMN and LMN signs in two regions with some UMN signs rostral to the LMN signs
Clinically definite ALS	UMN and LMN signs in three regions

UMN: upper motor neuron and LMN: lower motor neuron.

## Data Availability

The data used to support the findings of this study are included within the article.

## Supplementary Materials

Supplementary Materials The search expressions are shown in Appendix 1.

## References

[1] Shin J.-Y., Lee K.-W. (2015). Diagnosis and management of amyotrophic lateral sclerosis. *Journal of the Korean Medical Association*.

[2] Rowland L. P. (2001). How amyotrophic lateral sclerosis got its name. *Archives of Neurology*.

[3] Walling A. (1999). Amyotrophic lateral sclerosis: Lou Gehrig’s disease. *American Family Physician*.

[4] Zhu J., Shen L., Lin X., Hong Y., Feng Y. (2017). Clinical research on traditional Chinese medicine compounds and their preparations for amyotrophic lateral sclerosis. *Biomedicine & Pharmacotherapy*.

[5] Logroscino G., Traynor B. J., Hardiman O. (2010). Incidence of amyotrophic lateral sclerosis in Europe. *Journal of Neurology, Neurosurgery & Psychiatry*.

[6] Longinetti E., Fang F. (2019). Epidemiology of amyotrophic lateral sclerosis: an update of recent literature. *Current Opinion in Neurology*.

[7] Cai M., Yang E. J. (2019). Complementary and alternative medicine for treating amyotrophic lateral sclerosis: a narrative review. *Integrative Medicine Research*.

[8] Pan W., Chen X., Bao J. (2013). The use of integrative therapies in patients with amyotrophic lateral sclerosis in Shanghai, China. *Evidence-based Complementary and Alternative Medicine*.

[9] Kim S., Mun S., Park J., Choi S., Lee S., Kim S. (2019). Complementary and alternative medicine use in amyotrophic lateral sclerosis cases in South Korea. *Evidence-based Complementary and Alternative Medicine*.

[10] Wasner M., Klier H., Borasio G. (2001). The use of alternative medicine by patients with amyotrophic lateral sclerosis. *Journal of the Neurological Sciences*.

[11] Arksey H., O’malley L. (2005). Scoping studies: towards a methodological framework. *International Journal of Social Research Methodology*.

[12] Lockwood C., Dos Santos K. B., Pap R. (2019). Practical guidance for knowledge synthesis: scoping review methods. *Asian Nursing Research*.

[13] Wu J. (1995). Treating nervous system diseases with Maqianzi. *New Journal of Traditional Chinese Medicine*.

[14] Kim T., Lee B., Jeon J., Lew J. (2000). A case of amyotrophic lateral sclerosis. *The Journal of Internal Korean Medicine*.

[15] Park B., Lee E., Ko H. (2001). A study on the efficiency of riluzole and oriental medical treatment in amyotrophic lateral sclerosis. *The Journal of Internal Korean Medicine*.

[16] Kim K., Jung S., Jang J., Shin Y. (2004). A case report on the patient with amyotrophic lateral sclerosis (ALS). *Journal of Korean Medicine Rehabilitation*.

[17] Yeon C., Pak H., Jo Y., Jung J., Lee S., Kim S. (2010). The clinical case of oriental medical treatment at tender point for patient with lower back pain suggesting of amyotrophic lateral sclerosis. *The Journal of Korea CHUNA Manual Medicine for Spine & Nerves*.

[18] Wang S., Yang X., Deng T., Liu X. (2010). Summary of professor Deng Tietao’s experience in treating amyotrophic lateral sclerosis. *Journal of Guangzhou University of Traditional Chinese Medicine*.

[19] Lian Y., Liu N., Zhao J. (2012). Treating motor neuron disease in TCM. *Clinical Journal of Chinese Medicine*.

[20] Jeong H. H., Kim S. H., Lee S. M. (2013). A case study on the use of trihexyphenidyl, Korean medical treatment for the control of sialorrhea in patients with amyotrophic lateral sclerosis (ALS). *The Acupuncture*.

[21] Jeong H. H., Lee S. M., Lee J. C., Park M. Y., Song B. K., Kim S. C. (2013). A case study on the use of megestrol acetate and Korean medical treatment for the loss of appetite and weight loss in patients with amyotrophic lateral sclerosis (ALS). *The Acupuncture*.

[22] Lee J. C., Jeong H. H., Cha E. H. (2014). A clinical case study on the long term respiration management of amyotrophic lateral sclerosis patient with respiratory failure. *The Acupuncture*.

[23] Jo C., Huh J., Jang S., Ahn H. (2014). Case of amyotrophic lateral sclerosis (ALS) with oriental medical treatment evaluated by K-ALSFRS-R. *The Journal of Korean Academy of Medical Gi-gong*.

[24] Sun Y., Xu N. (2014). Jiaji electroacupuncture combined with traditional Chinese medicine to improve amyotrophic lateral sclerosis: a case. *Journal of Clinical Acupuncture and Moxibustion*.

[25] Zhong S., Zhang J., Zhang X., Feng Y., Zhu X., Zhang Q. (2014). Three medical records of professor Zhang Jiemei’s treatment of difficult miscellaneous diseases. *Liaoning Journal of Traditional Chinese Medicine*.

[26] Lu Y., Zhao J. (2015). Zhao Jianguo’s treatment of a case of amyotrophic lateral sclerosis. *Hunan Journal of Traditional Chinese Medicine*.

[27] Kim S.-H., Lee M.-S., Park Y.-G., Bae N.-Y. (2016). A case study of a taeyangin patient with amyotrophic lateral sclerosis. *Journal of Sasang constitutional medicine*.

[28] Cha E. H., Lee S. J., Lee J. I., Song I. J., Kim S. C. (2016). A case report on the use of processed extract and Korean medical treatment for a patient with amyotrophic lateral sclerosis (ALS). *The Acupuncture*.

[29] Qiu H., Li J. H., Yin S. B., Ke J. Q., Qiu C. L., Zheng G. Q. (2016). Dihuang yinzi, a classical Chinese herbal prescription, for amyotrophic lateral sclerosis: a 12-year follow-up case report. *Medicine*.

[30] Cao L., Zhang Q., Qiu C. (2017). Clinical medication experience of professor QIU changlin in treating amyotrophic lateral sclerosis by identifying tongue manifestation. *China Modern Doctor*.

[31] Liu A., Zhu D., Li X. (2018). A case of acupuncture combined with traditional Chinese medicine for treatment of amyotrophic lateral sclerosis. *Hunan Journal of Traditional Chinese Medicine*.

[32] Liang M. (1999). Treatment of amyotrophic lateral sclerosis with Chinese medicine combined with point injection. *Henan Traditional Chinese Medicine*.

[33] Cheng Y. (1999). Clinical observation on 46 cases of amyotrophic lateral sclerosis treated from du meridian. *Zhejiang Journal of Integrated Traditional Chinese and Western Medicine*.

[34] Luo R., Liu Y., Liu X., Xiong W. (2002). Treatment of amyotrophic lateral sclerosis by TCM:A clinical observation of 26 cases. *New Journal of Traditional Chinese Medicine*.

[35] Kwon K. (2003). Clinical studies of amyotrophic lateral sclerosis (ALS) through Korean medicine. *The journal of Korean Acupuncture & Moxibustion Society*.

[36] Byun M., Kim J., Sim S. (2007). Three cases of amyotrophic lateral sclerosis treated with oriental medical therapy. *The Journal of Internal Korean Medicine*.

[37] Ryu M., Wi J., Bang S., Lee J., Kim J., Yun Y. (2009). 2 cases of amyotrophic lateral sclerosis (ALS) with oriental medical treatment evaluated by K-ALSFRS-R and ALSSS. *The Journal of Korean Acupuncture & Moxibustion Society*.

[38] Jeon Y., Moon S., Ko C. (1997). Clinical study on the ALS (amyotrophic lateral sclerosis) patients in the department of circulatory internal medicine of kyung hee oriental medical hospital. *The Journal of Internal Korean Medicine*.

[39] Li H., Chen J., Weng W. (2017). Clinical features of amyotrophic lateral sclerosis: a retrospective study of 288 cases. *Shanghai Journal of Traditional Chinese Medicine*.

[40] Liu Z. (2001). Clinical study on the law of syndrome differentiation and treatment.

[41] Liu Y., Liu Z., Song Y., Lan H. (2006). The onset characteristics of 40 patients with amyotrophic lateral sclerosis and the analysis of curative effect before and after TCM treatment. *Traditional Chinese Medicine Journal*.

[42] Kim S.-C., Na W.-M., Lim N.-R., Lee D.-S., Jang E.-H., Song B.-K. (2009). A pilot clinical study on the Traditional Korean Medicine treatment of Amyotrophic lateral sclerosis. *Journal of Korean Institute of Herbal Acupuncture*.

[43] Kim H.-S., Song B.-K., Park M.-Y., Lim N.-R., Kim S.-H., Kim S.-C. (2010). The follow-up study on patients of amyotrophic lateral sclerosis after 1 year. *Journal of Korean Pharmacopuncture Institute*.

[44] Sun L. (2009). The influence research of jian pi yi fei fa to amyotrophic lateral sclerosis’s motor function and TCM syndrome.

[45] Zhong Y. (2011). Patients with amyotrophic lateral sclerosis assessment and the prelimeinary study of jian pi yi fei fang to amyotrophic lateral sclerosis’s swallowing function.

[46] Li J. (2012). The treatment study of amyotrophic lateral sclerosis’s motor function and the quality of life based on the theory of lung and spleen.

[47] Luo X. (2012). The study of lung and spleen treatment to amyotrophic lateral sclerosis patients’ influence of the traditional chinese medicine symptoms points.

[48] Zhao J. (2013). Clinical curative effect observation of cable from the lung and spleen treatment to amyotrophic lateral sclerosis patients.

[49] Wu Y. (2015). Clinical research on the effectiveness of strengthening the kidney and spleen in treating motor neuron disease.

[50] Meng T. (2018). Qirong granules and Taiyi Shen acupuncture for motor neurone disease clinical efficacy observation.

[51] Wen L., Li Y., Shen J., Lin W. (2019). Clinical study on Jianpi yifei recipe combined with western medicine for treatment of amyotrophic lateral sclerosis with lung and spleen deficiency type. *Journal of Guangzhou University of Traditional Chinese Medicine*.

[52] Chin M., Zhou L., Zhang L. (2012). Treatment of 23 cases of amyotrophic lateral sclerosis complicated with respiratory failure and constipation with Chinese medicine and massage. *Shandong Journal of Traditional Chinese Medicine*.

[53] Jin P. (2013). Clinical observation on treating amyotrophic lateral sclerosis by Guilu Erxian glues. *Clinical Journal of Chinese Medicine*.

[54] Pan W., Su X., Bao J. (2013). Open randomized clinical trial on JWSJZ decoction for the treatment of ALS patients. *Evidence-based Complementary and Alternative Medicine*.

[55] Fang Z. (2016). Assesment of bulbar pulsy in amyotrophic lateral sclerosis patients and evaluation of the effect of Jianpiyifei decoction.

[56] Bao J., Fu M., Chen B., Zhu X. (2016). Clinical study on modified Sijunzi decoction in treating amyotrophic lateral sclerosis. *Herald of Medicine*.

[57] Zhu X., Zhang H., Li H. (2017). Clinical efficacy and safety evaluation of supplementary Sijunzi decoction in treatment of ALS patients with splenasthenic syndrome. *Chlinical Misdiagnosis & Mistherapy.*.

[58] Gao P., Liao W., Sun C., Jiang W., Pan W., Liu T. (2017). Traditional Chinese herbs improve salivation and frequent nighttime urination in patients with amyotrophic lateral sclerosis. *Integrative medicine international*.

[59] Yang M. (2020). Safety and effectiveness of Mecasin and riluzole combination treatment for amyotrophic lateral sclerosis; a randomized, double blind, placebo controlled, parallel group, Phase 2a Clinical Trial.

[60] Qiu C. (2007). Current status and prospect of TCM diagnosis and treatment of amyotrophic lateral sclerosis. *Zhejiang Journal of Integrated Traditional Chinese and Western Medicine*.

[61] Zhao Y. (2011). Research progress in treatment of amyotrophic lateral sclerosis (ALS) with traditional Chinese medicine. *Asia-Pacific Traditional Medicine*.

[62] Xu W., Qin Y., Wang Q., Zhang L., Zhou Y. (2015). Research progress on treatment of amyotrophic lateral sclerosis with traditional Chinese medicine. *China Pharmacy*.

[63] Deng W. (2016). Research progress of traditional Chinese medicine on amyotrophic lateral sclerosis (part 2). *Pharmacology and Clinics of Chinese Materia Medica*.

[64] Mao Y., Han G., Zhao H. (2017). A review on treating amyotrophic lateral sclerosis in TCM. *Clinical Journal of Chinese Medicine*.

[65] Li N., Zhan Q. (2018). Progress in research on amyotrophic lateral sclerosis in traditional Chinese medicine. *Journal of Neurology and Neurorehabilitation*.

[66] Kim K. M., Kim J. H., Kim Y. I., Jeon J. H. (2015). A literature review of Korean medical studies of amyotrophic lateral sclerosis (ALS). *The Acupuncture*.

[67] Lee H., Park H., Lee D., Lee I., Hong J., Kwon J. (2015). A review of Korean studies on amyotrophic lateral sclerosis treated with traditional Korean medicine. *The Journal of the Society of Stroke on Korean Medicine*.

[68] Li X., Liu M., Yin A., Han C., Liu Y., Liu J. (2016). Meta analysis on traditional Chinese medicine tonic class prescriptions in treatment of amyotrophic lateral sclerosis. *Chinese Journal of Experimental Traditional Medical Formulae*.

[69] Brooks B. R., Miller R. G., Swash M., Munsat T. L. (2000). El Escorial revisited: revised criteria for the diagnosis of amyotrophic lateral sclerosis. *Amyotrophic Lateral Sclerosis and Other Motor Neuron Disorders*.

[70] Cedarbaum J., Stambler N., Malta E. (1999). The ALSFRS-R: a revised ALS functional rating scale that incorporates assessments of respiratory function. *Journal of the Neurological Sciences*.

[71] Kim H., Park K., Koh S. (2007). Korean version of amyotrophic lateral sclerosis functional rating scale-revised: a pilot study on the reliability and validity. *Journal of the Korean Neurological Association*.

[72] Baek W., Park A., Kim H., Kim S. (2011). Amyotrophic lateral sclerosis in Korea: clinical characteristics and prognostic factors. *Journal of the Korean Medical Association*.

[73] Kimura F., Fujimura C., Ishida S. (2006). Progression rate of ALSFRS-R at time of diagnosis predicts survival time in ALS. *Neurology*.

[74] Kim S., Chung S. E., Lee S., Park J., Choi S., Kim S. (2016). Experience of complementary and alternative medicine in patients with amyotrophic lateral sclerosis and their families: a qualitative study. *Amyotrophic Lateral Sclerosis & Frontotemporal Degeneration*.

