# Supplementary material for: Traditional East Asian Herbal Medicine for Amyotrophic Lateral Sclerosis: A Scoping Review
Source: Evid Based Complement Alternat Med. 2021 Dec 6;2021:5674142. doi: 10.1155/2021/5674142 (PMC8668313; doi:10.1155/2021/5674142)
Supplement: Supplementary Materials — The search expressions are shown in Appendix 1. [file 5674142.f1.docx]

Appendix 1

[Pubmed]

1. ("Amyotrophic Lateral Sclerosis"[MeSH Terms]) OR ("Motor Neuron Disease"[MeSH Terms]) 27947
2. (((((((((((("Amyotrophic Lateral Sclerosis"[Title/Abstract]) OR ("Lou Gehrig Disease"[Title/Abstract])) OR ("Lou Gehrig's Disease"[Title/Abstract])) OR ("Lou Gehrigs Disease"[Title/Abstract])) OR ("Gehrig's Disease"[Title/Abstract])) OR ("Gehrig Disease"[Title/Abstract])) OR ("Gehrigs Disease"[Title/Abstract])) OR ("Charcot Disease"[Title/Abstract])) OR ("Lou-Gehrigs"[Title/Abstract])) OR ("Motor Neuron Diseases"[Title/Abstract])) OR ("Motor Neuron Disease"[Title/Abstract])) OR ("Motor System Disease"[Title/Abstract])) OR ("Motor System Diseases"[Title/Abstract]) 27094
3. 1 OR 2 37486
4. ((((("Herbal Medicine"[MeSH Terms]) OR ("Drugs, Chinese Herbal"[MeSH Terms])) OR ("Medicine, Korean Traditional"[MeSH Terms])) OR ("Medicine, East Asian Traditional"[MeSH Terms])) OR ("Medicine, Chinese Traditional"[MeSH Terms])) OR ("Medicine, Kampo"[MeSH Terms]) 61447
5. ((((((((((((((((("Herb"[Title/Abstract]) OR ("Herbal"[Title/Abstract])) OR ("Herbal Medicine"[Title/Abstract])) OR ("Traditional Medicine"[Title/Abstract])) OR ("Oriental Medicine"[Title/Abstract])) OR ("East Medicine"[Title/Abstract])) OR ("East Medicines"[Title/Abstract])) OR ("East Asia Medicine"[Title/Abstract])) OR ("East Asia Medicines"[Title/Abstract])) OR ("Chinese Herbal"[Title/Abstract])) OR ("Chinese Plant Extracts"[Title/Abstract])) OR ("Chinese Plant Extract"[Title/Abstract])) OR ("Chinese Drugs"[Title/Abstract])) OR ("Chinese Drug"[Title/Abstract])) OR ("Korea Medicine"[Title/Abstract])) OR ("Korean Medicine"[Title/Abstract])) OR ("Kampo"[Title/Abstract])) OR ("Kanpo"[Title/Abstract]) 63063
6. 4 OR 5 109170
7. 3 AND 6 57
8. Up to December 31, 2019 50

[Embase]

1. 'amyotrophic lateral sclerosis'/exp OR 'motor neuron disease'/exp 47204
2. 'amyotrophic lateral sclerosis':ab,ti OR 'lou gehrig disease':ab,ti OR 'lou gehrigs disease':ab,ti OR 'gehrig disease':ab,ti OR 'gehrigs disease':ab,ti OR 'charcot disease':ab,ti OR 'lou-gehrigs':ab,ti OR 'motor neuron diseases':ab,ti OR 'motor neuron disease':ab,ti OR 'motor system disease':ab,ti OR 'motor system diseases':ab,ti 36298
3. 1 OR 2 51817
4. 'herbal medicine'/exp OR 'chinese medicine'/exp OR 'korean medicine'/exp OR 'oriental medicine'/exp OR 'chinese drug'/exp OR 'kampo medicine'/exp OR 'chinese medicinal formula'/exp 73151
5. 'herb':ab,ti OR 'herbal':ab,ti OR 'herbal medicine':ab,ti OR 'traditional medicine':ab,ti OR 'oriental medicine':ab,ti OR 'east medicine':ab,ti OR 'east medicines':ab,ti OR 'east asia medicine':ab,ti OR 'east asia medicines':ab,ti OR 'chinese herbal':ab,ti OR 'chinese plant extracts':ab,ti OR 'chinese plant extract':ab,ti OR 'chinese drugs':ab,ti OR 'chinese drug':ab,ti OR 'korea medicine':ab,ti OR 'korean medicine':ab,ti OR 'kampo':ab,ti OR 'kanpo':ab,ti OR 'chinese medicine':ab,ti OR 'kampo medicine':ab,ti OR 'chinese medicinal formula':ab,ti 117535
6. 4 OR 5 148774
7. 3 AND 6 127
8. Up to December 31, 2019 116

[Cochrane]

1. MeSH descriptor: [Amyotrophic Lateral Sclerosis] explode all trees 550
2. MeSH descriptor: [Motor Neuron Disease] explode all trees 697
3. #1 or #2 697
4. ("Amyotrophic Lateral Sclerosis" OR "Lou Gehrig Disease" OR "Lou Gehrig's Disease" OR "Lou Gehrigs Disease" OR "Gehrig's Disease" OR "Gehrig Disease" OR "Gehrigs Disease" OR "Charcot Disease" OR "Lou-Gehrigs" OR "Motor Neuron Diseases" OR "Motor Neuron Disease" OR "Motor System Disease" OR "Motor System Diseases"):ti,ab,kw (Word variations have been searched) 1354
5. #3 or #4 1440
6. MeSH descriptor: [Herbal Medicine] explode all trees 60
7. MeSH descriptor: [Drugs, Chinese Herbal] explode all trees 3524
8. MeSH descriptor: [Medicine, Korean Traditional] explode all trees 31
9. MeSH descriptor: [Medicine, East Asian Traditional] explode all trees 1254
10. MeSH descriptor: [Medicine, Chinese Traditional] explode all trees 1141
11. MeSH descriptor: [Medicine, Kampo] explode all trees 44
12. #6 or #7 or #8 or #9 or #10 or #11 4251
13. ("Herb" OR "Herbal" OR "Herbal Medicine" OR "Traditional Medicine" OR "Oriental Medicine" OR "East Medicine" OR "East Medicines" OR "East Asia Medicine" OR "East Asia Medicines" OR "Chinese Herbal" OR "Chinese Plant Extracts" OR "Chinese Plant Extract" OR "Chinese Drugs" OR "Chinese Drug" OR "Korea Medicine" OR "Korean Medicine" OR "Kampo" OR "Kanpo"):ti,ab,kw (Word variations have been searched) 11550
14. #12 or #13 12125
15. #5 and #14 17
16. Up to December 31, 2019 17

[Scopus]

1. INDEXTERMS ( "Amyotrophic Lateral Sclerosis"  OR  "Motor Neuron Disease" )  38031
2. TITLE-ABS-KEY ( "Amyotrophic Lateral Sclerosis"  OR  "Lou Gehrig Disease"  OR  "Lou Gehrig's Disease"  OR  "Lou Gehrigs Disease"  OR  "Gehrig's Disease"  OR  "Gehrig Disease"  OR  "Gehrigs Disease"  OR  "Charcot Disease"  OR  "Lou-Gehrigs"  OR  "Motor Neuron Diseases"  OR  "Motor Neuron Disease"  OR  "Motor System Disease"  OR  "Motor System Diseases" )  44720
3. 1 OR 2 44720
4. INDEXTERMS ( "Herbal Medicine" OR "Drugs, Chinese Herbal" OR "Medicine, Korean Traditional" OR "Medicine, East Asian Traditional" OR "Medicine, Chinese Traditional" OR "Medicine, Kampo" ) 70449
5. TITLE-ABS-KEY ( "Herb" OR "Herbal" OR "Herbal Medicine" OR "Traditional Medicine" OR "Oriental Medicine" OR "East Medicine" OR "East Medicines" OR "East Asia Medicine" OR "East Asia Medicines" OR "Chinese Herbal" OR "Chinese Plant Extracts" OR "Chinese Plant Extract" OR "Chinese Drugs" OR "Chinese Drug" OR "Korea Medicine" OR "Korean Medicine" OR "Kampo" OR "Kanpo" ) 216803
6. 4 OR 5 223933
7. 3 AND 6 123
8. Up to December 31, 2019 108

[CNKI]

1. (TI="肌萎缩性脊髓侧索硬化症"+"肌萎缩侧索硬化症"+"肌萎缩性侧索硬化症"+"肌萎缩侧索硬化"+"乙酰乳酸合成酶"+"Amyotrophic Lateral Sclerosis"+"Lou Gehrig Disease"+"Lou Gehrig's Disease"+"Lou Gehrigs Disease"+"Gehrig's Disease"+"Gehrig Disease"+"Gehrigs Disease"+"Charcot Disease"+"Lou-Gehrigs"+"Motor Neuron Diseases"+"Motor Neuron Disease"+"Motor System Disease"+"Motor System Diseases") OR (AB="肌萎缩性脊髓侧索硬化症"+"肌萎缩侧索硬化症"+"肌萎缩性侧索硬化症"+"肌萎缩侧索硬化"+"乙酰乳酸合成酶"+"Amyotrophic Lateral Sclerosis"+"Lou Gehrig Disease"+"Lou Gehrig's Disease"+"Lou Gehrigs Disease"+"Gehrig's Disease"+"Gehrig Disease"+"Gehrigs Disease"+"Charcot Disease"+"Lou-Gehrigs"+"Motor Neuron Diseases"+"Motor Neuron Disease"+"Motor System Disease"+"Motor System Diseases") 34106
2. (TI="草药"+"中草药"+"中药材"+"中药"+"中医药"+"中药治疗"+"韩国草药"+"韩药"+"汉方"+"汉方医学"+"日本汉方医学"+"Herb"+"Herbal"+"Herbal Medicine"+"Traditional Medicine"+"Oriental Medicine"+"East Medicine"+"East Medicines"+"East Asia Medicine"+"East Asia Medicines"+"Chinese Herbal"+"Chinese Plant Extracts"+"Chinese Plant Extract"+"Chinese Drugs"+"Chinese Drug"+"Korea Medicine"+"Korean Medicine"+"Kampo"+"Kanpo") OR (AB="草药"+"中草药"+"中药材"+"中药"+"中医药"+"中药治疗"+"韩国草药"+"韩药"+"汉方"+"汉方医学"+"日本汉方医学"+"Herb"+"Herbal"+"Herbal Medicine"+"Traditional Medicine"+"Oriental Medicine"+"East Medicine"+"East Medicines"+"East Asia Medicine"+"East Asia Medicines"+"Chinese Herbal"+"Chinese Plant Extracts"+"Chinese Plant Extract"+"Chinese Drugs"+"Chinese Drug"+"Korea Medicine"+"Korean Medicine"+"Kampo"+"Kanpo") 883363
3. 1 AND 2 193
4. Up to December 31, 2019 181

[CiNii]

1. 筋萎縮性側索硬化症 OR "運動ニューロン疾患" OR "筋萎縮性側索硬化" OR "Amyotrophic Lateral Sclerosis" OR "Lou Gehrig Disease" OR "Lou Gehrig's Disease" OR "Lou Gehrigs Disease" OR "Gehrig's Disease" OR "Gehrig Disease" OR "Gehrigs Disease" OR "Charcot Disease" OR "Lou-Gehrigs" OR "Motor Neuron Diseases" OR "Motor Neuron Disease" OR "Motor System Disease" OR "Motor System Diseases" 3722
2. "草薬" OR "中草薬" OR "中医薬" OR "中薬" OR "韓薬" OR "韓方薬" OR "漢方薬" OR "漢方" OR "漢方医学" OR "Herb" OR "Herbal" OR "Herbal Medicine" OR "Traditional Medicine" OR "Oriental Medicine" OR "East Medicine" OR "East Medicines" OR "East Asia Medicine" OR "East Asia Medicines" OR "Chinese Herbal" OR "Chinese Plant Extracts" OR "Chinese Plant Extract" OR "Chinese Drugs" OR "Chinese Drug" OR "Korea Medicine" OR "Korean Medicine" OR "Kampo" OR "Kanpo" 38953
3. 1 AND 2 10
4. Up to December 31, 2019 10

[NDSL]

1. (근위축성측삭경화증|근위축성측색경화증|루게릭병|"Amyotrophic Lateral Sclerosis"|"Lou Gehrig Disease"|"Motor Neuron Disease") (한약|한의학|중의학|중약|동양의학|전통의학|한방|"Herb"|"Herbal Medicine"|"Traditional Chinese Medicine"|"Kampo") 108
2. Up to December 31, 2019 105

[OASIS]

1. 근위축성측삭경화증 OR 근위축성측색경화증 OR 루게릭병 OR Amyotrophic Lateral Sclerosis OR Lou Gehrig Disease 26
2. Up to December 31, 2019 26
